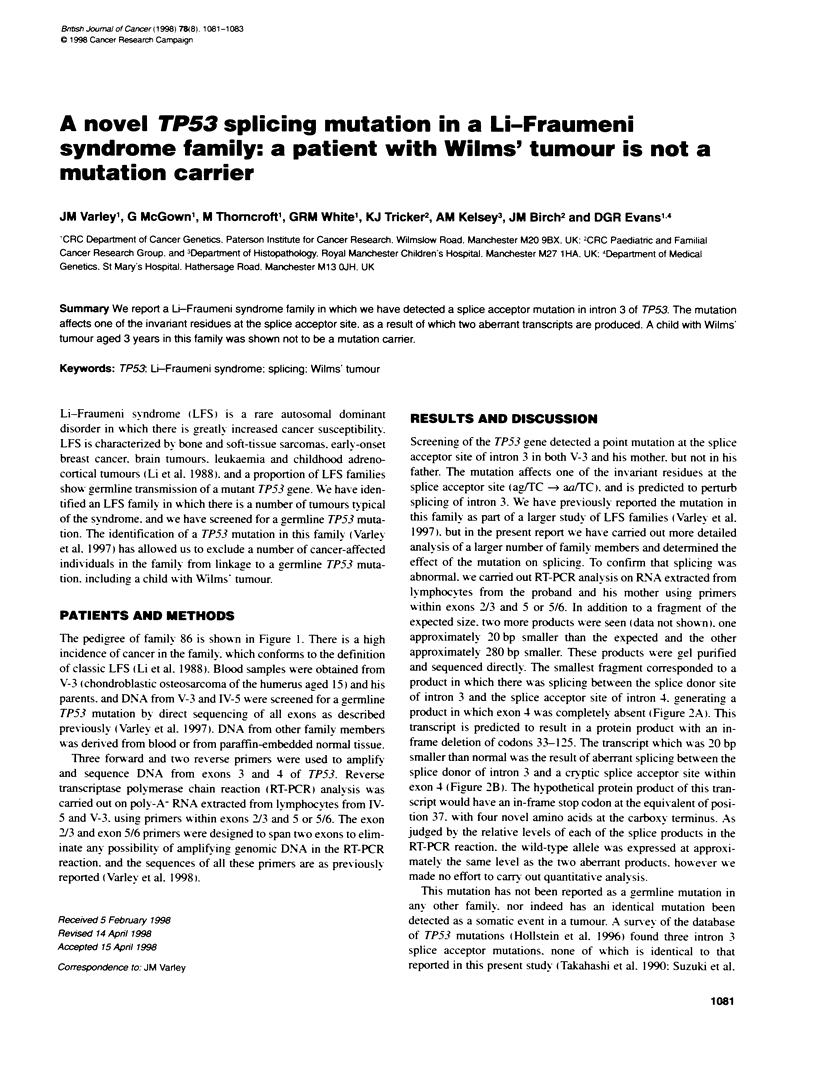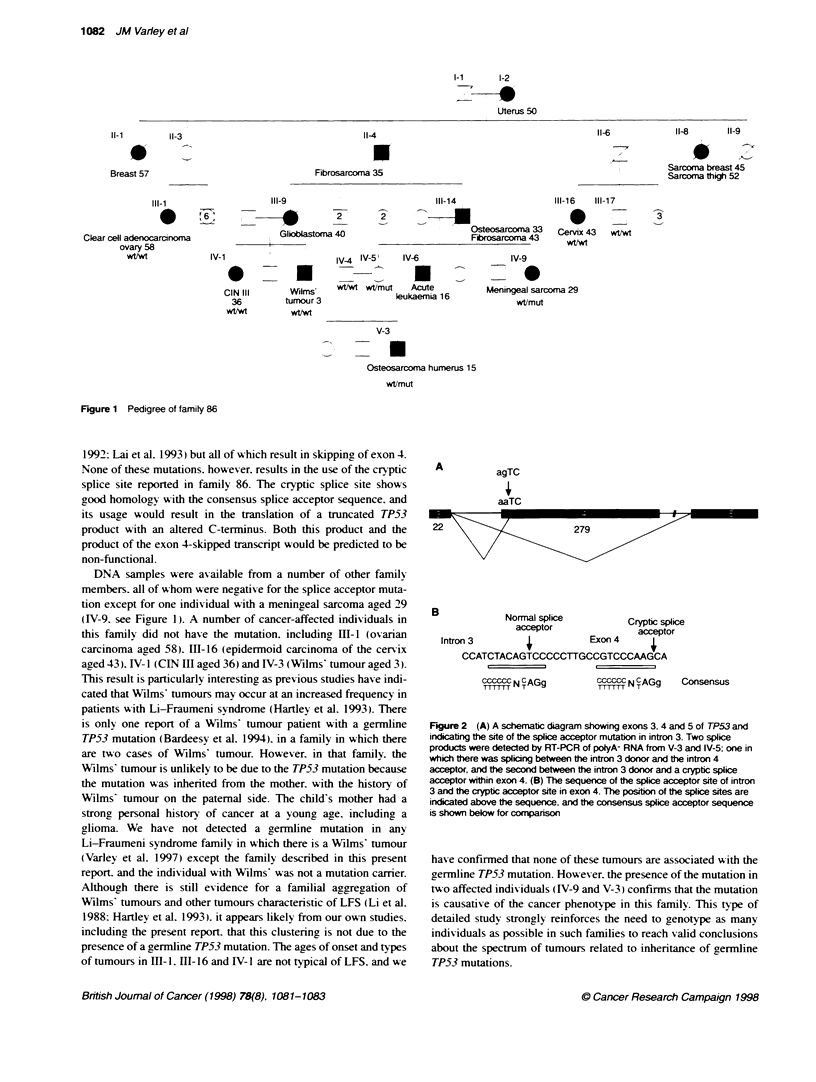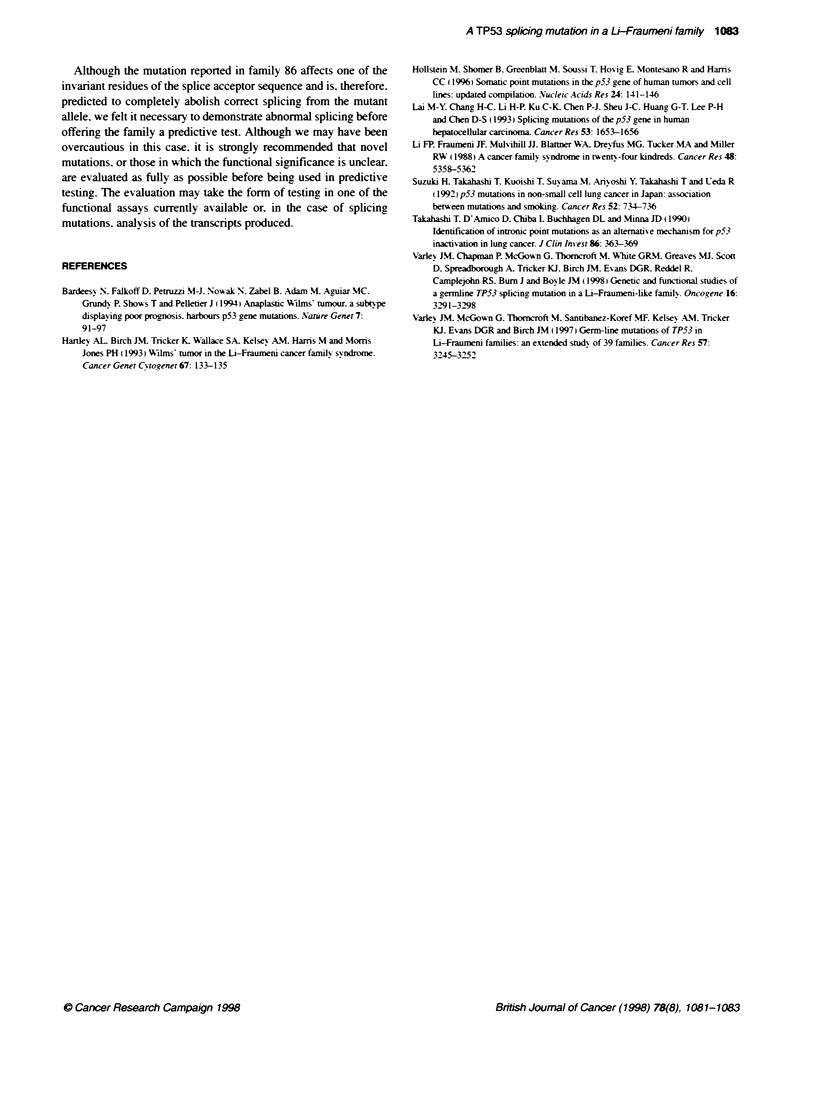# A novel TP53 splicing mutation in a Li-Fraumeni syndrome family: a patient with Wilms' tumour is not a mutation carrier.

**DOI:** 10.1038/bjc.1998.631

**Published:** 1998-10

**Authors:** J. M. Varley, G. McGown, M. Thorncroft, G. R. White, K. J. Tricker, A. M. Kelsey, J. M. Birch, D. G. Evans

**Affiliations:** CRC Department of Cancer Genetics, Paterson Institute for Cancer Research, Manchester, UK.

## Abstract

We report a Li-Fraumeni syndrome family in which we have detected a splice acceptor mutation in intron 3 of TP53. The mutation affects one of the invariant residues at the splice acceptor site, as a result of which two aberrant transcripts are produced. A child with Wilms' tumour aged 3 years in this family was shown not to be a mutation carrier.


British Journal of Cancer (1998) 78(8), 1081–1083
© 1998 Cancer Research Campaign

### A novel *TP53* splicing mutation in a Li–Fraumeni syndrome family: a patient with Wilms' tumour is not a mutation carrier

**JM Varley[1], G McGown[1], M Thorncroft[1], GRM White[1], KJ Tricker[2], AM Kelsey[3], JM Birch[2] and DGR Evans[1,4]**

[1]CRC Department of Cancer Genetics, Paterson Institute for Cancer Research, Wilmslow Road, Manchester M20 9BX, UK; [2]CRC Paediatric and Familial Cancer Research Group, and [3]Department of Histopathology, Royal Manchester Children's Hospital, Manchester M27 1HA, UK; [4]Department of Medical Genetics, St Mary's Hospital, Hathersage Road, Manchester M13 0JH, UK

**Summary** We report a Li–Fraumeni syndrome family in which we have detected a splice acceptor mutation in intron 3 of *TP53*. The mutation affects one of the invariant residues at the splice acceptor site, as a result of which two aberrant transcripts are produced. A child with Wilms' tumour aged 3 years in this family was shown not to be a mutation carrier.

**Keywords:** *TP53*; Li–Fraumeni syndrome; splicing; Wilms' tumour

Li–Fraumeni syndrome (LFS) is a rare autosomal dominant disorder in which there is greatly increased cancer susceptibility. LFS is characterized by bone and soft-tissue sarcomas, early-onset breast cancer, brain tumours, leukaemia and childhood adrenocortical tumours (Li et al, 1988), and a proportion of LFS families show germline transmission of a mutant *TP53* gene. We have identified an LFS family in which there is a number of tumours typical of the syndrome, and we have screened for a germline *TP53* mutation. The identification of a *TP53* mutation in this family (Varley et al, 1997) has allowed us to exclude a number of cancer-affected individuals in the family from linkage to a germline *TP53* mutation, including a child with Wilms' tumour.

### PATIENTS AND METHODS

The pedigree of family 86 is shown in Figure 1. There is a high incidence of cancer in the family, which conforms to the definition of classic LFS (Li et al, 1988). Blood samples were obtained from V-3 (chondroblastic osteosarcoma of the humerus aged 15) and his parents, and DNA from V-3 and IV-5 were screened for a germline *TP53* mutation by direct sequencing of all exons as described previously (Varley et al, 1997). DNA from other family members was derived from blood or from paraffin-embedded normal tissue.

Three forward and two reverse primers were used to amplify and sequence DNA from exons 3 and 4 of *TP53*. Reverse transcriptase polymerase chain reaction (RT-PCR) analysis was carried out on poly-$A^+$ RNA extracted from lymphocytes from IV-5 and V-3, using primers within exons 2/3 and 5 or 5/6. The exon 2/3 and exon 5/6 primers were designed to span two exons to eliminate any possibility of amplifying genomic DNA in the RT-PCR reaction, and the sequences of all these primers are as previously reported (Varley et al, 1998).

*Received 5 February 1998*
*Revised 14 April 1998*
*Accepted 15 April 1998*

*Correspondence to:* JM Varley

### RESULTS AND DISCUSSION

Screening of the *TP53* gene detected a point mutation at the splice acceptor site of intron 3 in both V-3 and his mother, but not in his father. The mutation affects one of the invariant residues at the splice acceptor site (ag/TC → *aa*/TC), and is predicted to perturb splicing of intron 3. We have previously reported the mutation in this family as part of a larger study of LFS families (Varley et al, 1997), but in the present report we have carried out more detailed analysis of a larger number of family members and determined the effect of the mutation on splicing. To confirm that splicing was abnormal, we carried out RT-PCR analysis on RNA extracted from lymphocytes from the proband and his mother using primers within exons 2/3 and 5 or 5/6. In addition to a fragment of the expected size, two more products were seen (data not shown), one approximately 20 bp smaller than the expected and the other approximately 280 bp smaller. These products were gel purified and sequenced directly. The smallest fragment corresponded to a product in which there was splicing between the splice donor site of intron 3 and the splice acceptor site of intron 4, generating a product in which exon 4 was completely absent (Figure 2A). This transcript is predicted to result in a protein product with an in-frame deletion of codons 33–125. The transcript which was 20 bp smaller than normal was the result of aberrant splicing between the splice donor of intron 3 and a cryptic splice acceptor site within exon 4 (Figure 2B). The hypothetical protein product of this transcript would have an in-frame stop codon at the equivalent of position 37, with four novel amino acids at the carboxy terminus. As judged by the relative levels of each of the splice products in the RT-PCR reaction, the wild-type allele was expressed at approximately the same level as the two aberrant products, however we made no effort to carry out quantitative analysis.

This mutation has not been reported as a germline mutation in any other family, nor indeed has an identical mutation been detected as a somatic event in a tumour. A survey of the database of *TP53* mutations (Hollstein et al, 1996) found three intron 3 splice acceptor mutations, none of which is identical to that reported in this present study (Takahashi et al, 1990; Suzuki et al,

**Figure 1** Pedigree of family 86

1992; Lai et al. 1993) but all of which result in skipping of exon 4. None of these mutations, however, results in the use of the cryptic splice site reported in family 86. The cryptic splice site shows good homology with the consensus splice acceptor sequence, and its usage would result in the translation of a truncated *TP53* product with an altered C-terminus. Both this product and the product of the exon 4-skipped transcript would be predicted to be non-functional.

DNA samples were available from a number of other family members, all of whom were negative for the splice acceptor mutation except for one individual with a meningeal sarcoma aged 29 (IV-9, see Figure 1). A number of cancer-affected individuals in this family did not have the mutation, including III-1 (ovarian carcinoma aged 58), III-16 (epidermoid carcinoma of the cervix aged 43), IV-1 (CIN III aged 36) and IV-3 (Wilms' tumour aged 3). This result is particularly interesting as previous studies have indicated that Wilms' tumours may occur at an increased frequency in patients with Li–Fraumeni syndrome (Hartley et al. 1993). There is only one report of a Wilms' tumour patient with a germline *TP53* mutation (Bardeesy et al. 1994), in a family in which there are two cases of Wilms' tumour. However, in that family, the Wilms' tumour is unlikely to be due to the *TP53* mutation because the mutation was inherited from the mother, with the history of Wilms' tumour on the paternal side. The child's mother had a strong personal history of cancer at a young age, including a glioma. We have not detected a germline mutation in any Li–Fraumeni syndrome family in which there is a Wilms' tumour (Varley et al. 1997) except the family described in this present report, and the individual with Wilms' was not a mutation carrier. Although there is still evidence for a familial aggregation of Wilms' tumours and other tumours characteristic of LFS (Li et al. 1988; Hartley et al. 1993), it appears likely from our own studies, including the present report, that this clustering is not due to the presence of a germline *TP53* mutation. The ages of onset and types of tumours in III-1, III-16 and IV-1 are not typical of LFS, and we have confirmed that none of these tumours are associated with the germline *TP53* mutation. However, the presence of the mutation in two affected individuals (IV-9 and V-3) confirms that the mutation is causative of the cancer phenotype in this family. This type of detailed study strongly reinforces the need to genotype as many individuals as possible in such families to reach valid conclusions about the spectrum of tumours related to inheritance of germline *TP53* mutations.

**Figure 2** (**A**) A schematic diagram showing exons 3, 4 and 5 of *TP53* and indicating the site of the splice acceptor mutation in intron 3. Two splice products were detected by RT-PCR of polyA⁻ RNA from V-3 and IV-5; one in which there was splicing between the intron 3 donor and the intron 4 acceptor, and the second between the intron 3 donor and a cryptic splice acceptor within exon 4. (**B**) The sequence of the splice acceptor site of intron 3 and the cryptic acceptor site in exon 4. The position of the splice sites are indicated above the sequence, and the consensus splice acceptor sequence is shown below for comparison

© Cancer Research Campaign 1998

Although the mutation reported in family 86 affects one of the invariant residues of the splice acceptor sequence and is, therefore, predicted to completely abolish correct splicing from the mutant allele, we felt it necessary to demonstrate abnormal splicing before offering the family a predictive test. Although we may have been overcautious in this case, it is strongly recommended that novel mutations, or those in which the functional significance is unclear, are evaluated as fully as possible before being used in predictive testing. The evaluation may take the form of testing in one of the functional assays currently available or, in the case of splicing mutations, analysis of the transcripts produced.

### REFERENCES

Bardeesy N, Falkoff D, Petruzzi M-J, Nowak N, Zabel B, Adam M, Aguiar MC, Grundy P, Shows T and Pelletier J (1994) Anaplastic Wilms' tumour, a subtype displaying poor prognosis, harbours p53 gene mutations. *Nature Genet* **7**: 91–97

Hartley AL, Birch JM, Tricker K, Wallace SA, Kelsey AM, Harris M and Morris Jones PH (1993) Wilms' tumor in the Li–Fraumeni cancer family syndrome. *Cancer Genet Cytogenet* **67**: 133–135

Hollstein M, Shomer B, Greenblatt M, Soussi T, Hovig E, Montesano R and Harris CC (1996) Somatic point mutations in the *p53* gene of human tumors and cell lines: updated compilation. *Nucleic Acids Res* **24**: 141–146

Lai M-Y, Chang H-C, Li H-P, Ku C-K, Chen P-J, Sheu J-C, Huang G-T, Lee P-H and Chen D-S (1993) Splicing mutations of the *p53* gene in human hepatocellular carcinoma. *Cancer Res* **53**: 1653–1656

Li FP, Fraumeni JF, Mulvihill JJ, Blattner WA, Dreyfus MG, Tucker MA and Miller RW (1988) A cancer family syndrome in twenty-four kindreds. *Cancer Res* **48**: 5358–5362

Suzuki H, Takahashi T, Kuoishi T, Suyama M, Ariyoshi Y, Takahashi T and Ueda R (1992) *p53* mutations in non-small cell lung cancer in Japan: association between mutations and smoking. *Cancer Res* **52**: 734–736

Takahashi T, D'Amico D, Chiba I, Buchhagen DL and Minna JD (1990) Identification of intronic point mutations as an alternative mechanism for *p53* inactivation in lung cancer. *J Clin Invest* **86**: 363–369

Varley JM, Chapman P, McGown G, Thorncroft M, White GRM, Greaves MJ, Scott D, Spreadborough A, Tricker KJ, Birch JM, Evans DGR, Reddel R, Camplejohn RS, Burn J and Boyle JM (1998) Genetic and functional studies of a germline *TP53* splicing mutation in a Li–Fraumeni-like family. *Oncogene* **16**: 3291–3298

Varley JM, McGown G, Thorncroft M, Santibanez-Koref MF, Kelsey AM, Tricker KJ, Evans DGR and Birch JM (1997) Germ-line mutations of *TP53* in Li–Fraumeni families: an extended study of 39 families. *Cancer Res* **57**: 3245–3252

© Cancer Research Campaign 1998